# Intention to take COVID-19 vaccine and associated factors among pregnant women attending antenatal care at public health facilities in Bahir Dar city, Northwest Ethiopia

**DOI:** 10.1186/s12905-023-02331-1

**Published:** 2023-04-11

**Authors:** Begizew Yimenu Mekuriaw, Dabere Nigatu, Anteneh Mengist Dessie, Melash Belachew Asresie

**Affiliations:** 1 Department of Midwifery, College of Health Science, Debre Tabor University, Debre Tabor, Ethiopia; 2 Department of Reproductive Health and Population Studies, School of Public Health, College of Medicine and Health Sciences, Bahir Dar University, Bahir Dar, Ethiopia; 3 Department of Public Health, College of Health Science, Debre Tabor University, Debre Tabor, Ethiopia

**Keywords:** COVID-19 vaccine, Bahir Dar, Intention, Northwest Ethiopia, Pregnant women

## Abstract

**Background:**

Pregnant mothers are a risky population group for COVID-19 and pregnant mothers with COVID-19 are at increased risk of hospitalization, intensive-care unit admission, invasive ventilation support, and maternal mortality. Vaccination is an essential tool in stopping the effect of the pandemic on maternal and child health. However, there are only limited studies in Ethiopia on the intention to take the COVID-19 vaccine among pregnant women. Thus, this study aimed to assess intention to take the COVID-19 vaccine and associated factors among pregnant women in Bahir Dar city, Northwest Ethiopia.

**Methods:**

Facility based cross-sectional study was conducted among 590 pregnant women from 23 May to 07 July 2022. The study participants were selected using a systematic sampling technique. Interviewer administrative questionnaire with epicollect5 application was used to collect the data. Both bi-variable and multivariable binary logistic regression analysis was performed. Statistical significance was defined at a 95% CI with a p-value < 0.05.

**Result:**

Overall, 19.8% (95% CI: 16.60–23.06%) of pregnant women intend to take the COVID-19 vaccine. Being urban residence (AOR = 3.40, 95% CI: 1.71–6.78), third trimester of gestational age (AOR = 3.11, 95% CI: 1.61–6.03), multipara (AOR = 2.30, 95% CI: 1.33–3.97), knowledge of COVID-19 vaccine (AOR = 2.33, 95% CI: 1.44–3.77) and having good attitude towards COVID-19 vaccine (AOR = 2.68, 95% CI: 1.65–4.33) were significantly associated with intention to take COVID-19 vaccine.

**Conclusion:**

In conclusion, the pregnant women’s intention to take the COVID-19 vaccine in this study area was very low. It was significantly associated with residency, gestational age, parity, knowledge, and attitude toward the vaccine. Therefore, strengthening interventions that improve knowledge and attitude about the COVID-19 vaccine, predominantly among those primipara mothers and mothers from rural residences, may raise the intention to take it.

**Supplementary Information:**

The online version contains supplementary material available at 10.1186/s12905-023-02331-1.

## Background

The coronavirus disease 2019 (COVID-19) vaccine intention measures a person’s readiness to receive the shot and their level of commitment to doing so [1]. Vaccine hesitancy was defined by the World Health Organization (WHO) Strategic Advisory Group of Experts as delay in accepting or refusing immunization despite the availability of vaccination services [2]. After the March 2020 declaration of the COVID-2019 pandemic by WHO [3], scientists and pharmaceutical companies are racing against time in efforts to develop vaccines [4, 5], since one key strategy in the global response to end the global COVID-19 pandemic is vaccination. The approaches to vaccine development were conventional whole virus vaccines (live attenuated or inactivated vaccines), recombinant protein-based vaccines (protein subunit vaccines, virus-like particles), viral vector vaccines, and nucleic acid vaccines (DNA and mRNA vaccines) [6].

Since the beginning of the COVID-19 outbreak, many COVID-19 vaccine candidates have entered clinical trials in less than 6 months and have been conditionally licensed in less than 10 months [7]. WHO has set a goal of reaching 70% COVID-19 vaccine coverage in all countries by the end of June 2022, in order to change the pandemic’s trajectory [8]. To achieve this goal: elders, health workers, and high-risk groups of all age was the first targeted populations to be vaccinated against COVID-19 [8]. Pregnant women are one of high risk population facing serious morbidities and mortality from COVID-19 who needs vaccine [9].

Thousands of individuals in Ethiopia have been impacted by the pandemic, and they are either sick or have died because of the disease’s spread. Pregnant women were at higher risk of serious illness caused by COVID-19, so its impact was more profound on them. To avert this problem, the Ethiopian Ministry of Health first introduced the COVID-19 vaccine on March 13, 2021, and then launched a COVID-19 vaccination campaign on November 16, 2021. More than 6.2 million COVID-19 vaccines were ready for the campaign. All of the vaccines (Sinopharm, AstraZeneca, Johnson & Johnson/Janssen, and Pfizer-BioNTech) had Ethiopian approval [10] and were safe for use during pregnancy and breastfeeding [11–13]. Through this campaign, the Ministry was transmitting information and calls to be vaccinated through short messaging system, different radio stations, and national television. However, as of 21 January 2023, only 53,514,115 vaccine doses have been administered [14].

However, vaccination program’s attainment depends on vaccine acceptability of the individuals including pregnant women [15]. WHO has discussed a number of strategies to combat vaccine hesitancy, including the involvement of community leaders, social mobilization techniques, mass media campaigns, the use of reminder and follow-up systems, training and education of healthcare professionals, nonfinancial incentives, vaccine mandates, efforts to make vaccination more convenient, and efforts to increase general knowledge and awareness about vaccines and vaccination [16].

The magnitude of intention to take COVID-19 vaccine among pregnant women varies from continent to continent. Systematic review and meta-analysis on 32 countries tell us, 54% of pregnant mothers were interested to be vaccinated against COVID-19 [17]. The highest acceptance rate was recorded in Asian countries ranging from 30 to 86.6% [18–22] while lowest was in Africa with 19% [23]. Studies in Ethiopia also revealed that COVID-19 vaccine acceptance among pregnant women were ranges from 18% in Debre Markos to 70.7% in southern Ethiopia [24–27].

Although there have been some studies in Ethiopia on pregnant women’s intentions to receive the COVID-19 vaccine, the results of these studies were inconsistent, both in terms of the percentage of pregnant women who have this intention and the factors influencing it [24–27]. This indicated that pregnant mother’s intention and contributing variables would vary across the nation, necessitating area-specific research to develop an effective evidence-based intervention. In addition, some variables such as trust in traditional medicine and home remedies, family influence, and experience of COVID-19 disease were not included in the previous studies [24–27], despite the fact that they are crucial to assessing COVID-19 vaccine acceptance. In light of these circumstances, the purpose of this study was to determine the intention of pregnant women to receive the COVID-19 vaccine and to pinpoint the key influencing variables.

## Methods

### Study design, period and area

An institution-based cross-sectional study was conducted among pregnant women who attend ANC at public health facilities in Bahir Dar city from 23 May to 07 July 2022. Bahir Dar is the capital city of the Amhara National Regional State in the Federal Democratic Republic of Ethiopia. According to Bahir Dar city administration plan commission 2014 report, the population of Bahir Dar city is estimated to be 406,433. Among these, 135,708 of them were reproductive age females [28]. The city has one specialized, one referral, and one primary public hospital (Tibebe Ghion, Felege Hiwot, and Addis Alem respectively), 10 health centers, 10 health posts, and one family guidance association clinic, 4 private general hospitals, and 35 medium private clinics.

### Study population and eligibility criteria

The study population of this study consisted of all pregnant women who visited ANC clinics at public health facilities during the study period; those who were seriously ill and/or who had received a vaccination during the period of data collection were excluded from the study.

### Sample size and sampling procedure

The minimum required sample size was calculated by using a single population proportion formula with the assumption of the proportion of pregnant mothers who have an intention to take COVID-19 vaccine (p = 40.08%) [25], 95% confidence level, and 5% marginal error. The sample required becomes 371 and final sample size after considering 1.5 design effect and 10% non-response rate was 612. Multi stage sampling techniques was used to reach study participants. There are 10 health centers and 3 hospitals from Bahir dar city administration. From these public health facilities, 5 health centers and 1 hospital were selected. The sample was allocated proportionally to each selected public health facilities based on the number of ANC attendees on follow-up at each selected health facilities one month preceding the survey. Finally, systematic random sampling was applied to select the study participants.

### Operational definitions

*Intention to take COVID-19 vaccine*: it was measured by asking respondents a single question whether they intended to take COVID-19 vaccines at this moment or during this pregnancy. Response options have 5 likert scales (from strongly agree 1 to strongly disagree 5) then it was dichotomized into two (strongly agree and agree labeled as 1 and from neutral to strongly disagree labeled as 0). Finally respondents who score 1 from one of the two questions were considered as have intention to take COVID-19 vaccine others have no intention to take it [15, 24–26, 29].

*Knowledge of the COVID-19 vaccine*: it was measured by five items, and it was analyzed as a binary variable. Participants who had correctly answered three and more questions will be considered as having “Good knowledge,” otherwise “Poor knowledge” [24, 25].

*Attitude toward COVID-19 vaccine*: it was measured by eighteen items adapted from previous literature [30]. Responses were rated on a 5-point scale from 1 = “strongly agree” to 5 = “strongly disagree.” It was dichotomized into “poor attitude” and “good attitude” based on the threshold which was calculated using the demarcation threshold formula: ((total highest score _total lowest score)/2) + total lowest score then pregnant mothers who scored less than this value was considered as poor attitude and those who scored greater than or equal to this value was considered as good attitude [24, 31].

### Data collection tools and procedures

The tool is adapted from other literatures [25, 28, 32]. The tool has five sections: the first section is about socio-demographic characteristics of the study participants, the second section is about obstetric and clinical characteristics of the participants, the third one is information and experience about COVID-19, the fourth one is about participants’ knowledge and attitude of the COVID-19 vaccine and last section inquires women’s intention to take COVID-19 vaccine. Pre-test was conducted a week before the start of actual data collection at Debre tabor comprehensive specialized hospital on 30 individuals (5% of the sample size). The data was collected by three BSc midwives with face to face interview using epicollect5 application with smart phone. To differentiate vaccinated and unvaccinated pregnant women, health care providers working in ANC was asked to label 1 for vaccinated and 0 for unvaccinated from their appointment card and order them to show for data collectors.

### Data processing and analysis

Data was entered in EpiData and exported to SPSS version 25 for analysis. Descriptive statistics: proportions, frequencies, standard deviations and mean were calculated and the findings of the analysis were presented in text, tables, and graphs. Binary logistic regression was run to see the association of each independent variable with COVID-19 vaccine acceptance and to select candidate variables to multi-variable logistic regression analysis. Variables with p-value < 0.25 in bi-variable logistic regression analysis were entered into multivariable logistic regression [33] and analyzed with backward stepwise method. To check the fitness of regression model, Hosmer and lemeshow test was performed and it was 0.634. Finally, significant factors were identified based on 95% confidence level, Adjusted Odd Ratio (AOR) and p-value < 0.05.

## Results

### Socio demographic characteristics

A total of 590 pregnant women were participated in the study with 96.4% of response rate. The mean age of the pregnant women was 26.94 ± 5.79.Most of pregnant women 544 (92.2%) were Amhara in their Ethnicity. 531(90%) and 440(74.6%) of pregnant women were married and live in urban areas respectively (Table 1).

**Table 1:** Socio-demographic characteristics of pregnant women attending ANC in Bahir Dar city public health facilities, North West Ethiopia (n = 590)

Variables	Categories	Frequency	Percent
Age	15–19	28	4.7
	20–24	215	36.4
	25–29	179	30.3
	30–34	84	14.2
	35 and above	84	14.2
Ethnicity	Amhara	544	92.2
	Tigrie	28	4.7
	Gurage and Oromo	18	3.1
Religion	Orthodox	474	80.3
	Muslim	77	13.1
	Protestant	24	4.1
	Catholic	15	2.5
Marital status	Married	531	90
	Never married	49	8.3
	Divorced	10	1.7
Mother educational status	Unable to read and write	108	18.3
	Able to read and write only	83	14.1
	Primary school (1–8)	157	26.6
	Secondary school (9–12)	127	21.5
	College and above	115	19.5
Partner educational status	Unable to read and write	88	14.9
	Able to read and write only	105	17.8
	Primary school (1–8)	101	17.1
	Secondary school (9–12)	143	24.2
	College and above	153	25.9
Mother occupation	Merchant	96	16.3
	Government employee	66	11.2
	Private employee	52	8.8
	Had no job	200	33.9
	Farmer	138	23.4
	Student and daily laborer	38	6.4
Residence	Urban	440	74.6
	Rural	150	25.4

### Obstetrics and clinical characteristics

In this study, 328(55.6%) of respondents were multigravida. From the total of 590 pregnant women, 268(45.4%) of them were in the 2nd trimester during the survey. Among the study participants, 37(6.3%) and 40(6.8%) of them had pregnancy related and chronic health problems respectively (Table 2).

**Table 2:** Obstetrics and clinical characteristics of pregnant women attending ANC in Bahir Dar city public health facilities, North West Ethiopia (n = 590)

Variables	Categories	Frequency	Percent
Gravidity	Primgravida	262	44.4
	Multigravida	328	55.6
Parity	Nully para	281	47.6
	Primipara	121	20.5
	Multi para	188	31.9
Trimesters	First	143	24.2
	Second	268	45.4
	Third	179	30.3
Number of ANC visit	One	255	43.2
	Two	158	26.8
	Three	89	15.1
	Four & above	88	14.9
Planned pregnancy	Yes	437	74.1
	No	153	25.9
Have health problem	Yes	37	6.3
	No	553	93.7
Problems with current pregnancy	PIH	18	3
	Gestational DM	10	1.69
	Anemia	11	1.86
	Others*	3	0.5
History of chronic disease	Yes	40	6.8
	No	550	93.2
Type of chronic disease	Hypertension	15	2.5
	Diabetes mellitus	14	2.3
	HIV AIDDS	11	1.9
	Others**	5	0.84
Having children	Yes	299	50.7
	No	291	49.3

*=*fetal movement decrement, hyperemesis gravidarum*

**=*asthma, cardiac and kidney problems*

### Knowledge and attitude on COVID-19 and its vaccine

In this study, all 590 (100%) of pregnant women had heard about COVID-19. Among pregnant mothers included in the study, 558 (94.6%) and 563 (95.4%) of them know at least one transmission and prevention methods of COVID-19, respectively. Only 226 (39.8%) and 142 (24.1%) of pregnant women had good knowledge and attitude, respectively. Out of 590 participants, 568 (96.3%) of them had heard about COVID-19 vaccine. However only 170(29.9%) of pregnant women knows that the vaccine can be given for them.

### Intention to take COVID-19 vaccine

This finding showed that only 117 (19.8%; 95% CI: 16.60–23.06) of pregnant women had an intention to take the COVID-19 vaccine. Of those, majority 105 (89.74%) mothers were from urban residences. Only 10 (8.55%) and 38 (32.48%) uneducated and nully para mothers were have an intention to take COVID-19 vaccine, respectively.

### Factors associated with intention to receive COVID-19 vaccine

Thirteen variables were checked in binary logistic regression analysis and all of them were entered in to multi-variable logistic regression. From thus five variables-namely; residence, gestational age, parity, knowledge and attitude on COVID-19 vaccine had significant association with the outcome variable. The odd of intending to take COVID-19 among urban pregnant women was 3.40 times higher compared to pregnant women from rural (AOR = 3.40, 95%CI: 1.71–6.78). Pregnant women who had good attitude toward COVID-19 vaccine were 2.68 times higher to take the vaccine than those who had poor attitude (AOR = 2.68, 95% CI: 1.65–4.33) (Table 3).

**Table 3:** Bi-variable and multi-variable logistic regression of pregnant women attending ANC in Bahir Dar city public health facilities, Northwest Ethiopia

Variables	Intention to take COVID-19 vaccine			COR(95% CI)	AOR(95% CI)
	Yes		No		
Educational status of the mother					
Unable to read and write	10	98		1	
Able to read and write only	11	72		1.40(0.60–3.71)	1.52(0.53–4.32)
Primary school (1–8)	27	130		2.03(0.94–4.40)	1.64(0.60–4.50)
Secondary school (9–12)	24	103		2.28(1.03–5.02)	1.59(0.56–4.48)
College and above	45	70		6.3(3-13.34)	3.27(1.06–10.04)
Educational status of the husband/partner					
Unable to read and write	10	78		1	1
Able to read and write only	15	90		1.3(0.55–3.05)	0.59(0.20–1.73)
Primary school (1–8)	14	87		1.25(0.52–2.98)	0.49(0.15–1.57)
Secondary school (9–12)	29	114		1.98(0.91–4.30)	0.58(0.18–1.82)
College and above	49	104		3.67(1.75–7.70)	0.70(0.21–2.32)
Occupation of the mother					
Have no job	33	167		1	1
Farmer	13	125		0.52(0.26–1.04)	2.63(0.73–9.41)
Merchant	30	66		2.30(1.30–4.07)	1.70(0.88–3.26)
Gov’t employee	24	42		2.89(1.54–5.40)	1.06(0.50–2.25)
Private employee	9	43		1.05(0.47–2.38)	0.68(0.27-1.71)
Others	8	30		1.34(0.56–3.20)	2.66(0.96–7.38)
Residence					
Rural	12	138		1	1
Urban	105	335		3.60(1.92–6.76)	3.40(1.71–6.78)*
Having children					
Yes	75	224		1.98(1.30–3.01)	0.48(0.12–1.87)
No	42	249		1	1
Parity					
Nully para	38	243		1	1
Primipara	25	96		1.66(0.95–2.90)	1.16(0.62–2.15)
Multipara	54	134		2.57(1.61–4.10)	2.30(1.33–3.97)*
Trimesters					
First	16	127		1	1
Second	33	235		1.11(0.59–2.10)	1.17(0.59–2.30)
Third	68	111		4.85(2.66–8.87)	3.11(1.61–6.03)*
Pregnancy planned					
Yes	97	338		1.93(1.15–3.26)	1.39(0.78–2.47)
No	20	135		1	1
Have health problems related with current pregnancy					
Yes	17	20		3.85(1.94–7.61)	1.52(0.64–3.57)
No	100	453		1	1
Family tested positive for COVID-19					
Yes	16	30		2.33(1.22–4.45)	1.01(0.44–2.30)
No	101	443		1	1
Knowing died due to COVID-19					
Yes	19	31		2.76(1.5–5.09	1.51(0.72–3.14)
No	98	442		1	1
Knowledge on COVID-19 vaccine					
Poor knowledge	39	325		1	1
Good knowledge	78	148		4.39(2.85–6.75)	2.33(1.44–3.77)*
Attitude towards COVID-19 vaccine					
Poor attitude	60	388		1	1
Good attitude	57	85		4.33(2.81–6.67)	2.68(1.65–4.33)*

Bold and * = P-value < 0.05, others = daily labors and students

## Discussion

The success of vaccination program depends on an intention of individuals to take vaccines. In order to accomplish this, studying the intention to take COVID-19 and associated factors is important. Therefore, this study tried to assess the intention to take the COVID-19 vaccine and associated factors among pregnant women, a risky population group. Accordingly, this study revealed that 19.8%% (95% CI: 16.60–23.06%) of pregnant women had intention to receive COVID-19 vaccine. This finding is similar with study conducted in Debre Markos 18% [27]. However, it was lower than other studies done in central Gondar zone 40.08% [25] and southern ethiopia 31.3% [24],70.7% [26]. The possible reason might be due to the difference in time periods between the studies. Since this study was done after the distribution of the vaccine for some population groups, it might create fear and a dilemma because pregnant mothers might have information on side effects from vaccinated people [34–36].

The result of this study is also lower than other studies conducted outside of Ethiopia including: Colombia 44.3% [37], United states 58.3% [38], Pennsylvania 58% [39], Czechia 76.6% [40], United kingdom 62.1% [29], a result of survey in 16 countries 52% [15], Switzerland 29.7%,turkey 70.4% [41], Ankara turkey37% [42], Japan 86.6% [20], China 77.4% [21], Qatar 75% [43], Thailand 60.8% [22], Vietnam 60.4% [19], Singapore 30.3% [18] and Australia 48% [44]. Possibly, it might be due to the sociodemographic difference of the participants. In contrast to this study, the participants in the aforementioned studies were mostly from developed countries, and they are highly educated, which in turn leads them to have higher intentions to take the COVID-19 vaccine [45]. Have a higher burden of COVID-19 in these countries will also make them more likely to have a greater intention to take the vaccine [46].

This finding reveals that residence, gestational age, parity, knowledge and attitude toward the vaccine had statistically significant association with intention to take COVID-19 vaccine. Pregnant women who lived in urban area were more likely to intend to take COVID-19 vaccine than rural ones. This finding is supported with other previous studies conducted in Ethiopia [24, 25] and Vietnam [19]. The possible justification for higher acceptance rate of COVID-19 vaccine among urban pregnant women could be due to the presence of media exposure. Another explanation might be the perception of COVID-19 susceptibility, as pregnant women in cities are more likely to live in crowded conditions and have higher thoughts of susceptibility, which in turn leads them to have a higher intention of taking the COVID-19 vaccine.

Gestational age was also one of the significant factors in this study, as in other previous studies done in the Czech Republic [40], China [21], Switzerland [47], and the UK [29]. Concerns on teratogenicity might be justification for low acceptance of COVID-19 vaccine in the first trimester of pregnancy. Parity was another factor that had a significant effect on the intention to take COVID-19 vaccine, which is supported by a French study [48]. Possibly multipara women will experience the advantage of childhood vaccines there by decide to take COVID-19 vaccine.

The result of our study showed that pregnant women who had good knowledge on COVID-19 vaccines were more likely to be vaccinated the vaccine than those who had poor knowledge. The finding was similar with studies conducted in southern Ethiopia [26] and Singapore [18].Pregnant women who had good knowledge might know the recommended and safe vaccines for them. In addition having good knowledge could help them not to disturb with rumors from the community there by decide timely. Therefore, they can decide to receive the vaccine without any hesitation.

The other most important factor which had significant association with intention be vaccinated for COVID-19 was attitude of pregnant women towards COVID-19 and its vaccine. Respondents who had good attitude toward the vaccine were more likely to receive COVID-19 vaccine compared with pregnant mothers who had poor attitude. This finding is supported by other studies done in southern Ethiopia [24], central Gondar zone [25], South Africa [49] and Saudi Arabia [50]. The possible reason might be that mothers with a good attitude may trust the scientific papers and guidelines of the country, so they can easily accept the vaccine.

### Strengths and limitations of the study

The study has a number of strengths. One of its strengths is that it was conducted in multiple health facilities of Bahir Dar city, which increases the representativeness of the findings for the source population. A face-to-face interview was also employed instead of an online survey and patient chart review so that we had more control over the factors and could get detailed information not available from other studies in Ethiopia. In addition, the desirability bias has been mitigated by the fact that participants were assured at the start of the interview that their responses would be fully anonymous. Despite many strengths, the study is not without limitations. Thus, the following limitations should be taken into account while interpreting the result: First, the cross-sectional nature of the study design does not permit establishing cause-and-effect relationships. Second, qualitative data supplementation was not there to explore socio-cultural factors that make pregnant mothers not have the intention of taking the COVID-19 vaccine. Third, there might be a possibility of social desirability bias due to interviewer-administered nature of the data collection process.

## Conclusion

The magnitude of intention to take COVID-19 vaccine was very low. The intention to take COVID-19 vaccine was associated with residence, gestational age of pregnancy, parity, knowledge and attitude towards the COVID-19 vaccine. This, to increase the uptake of COVID-19 vaccine in this risky population groups, it is important for FMOH, regional health bureaus and interested individuals to provide health education about the vaccine in order to develop the level of knowledge and attitude towards the COVID-19 vaccine. In addition giving emphasis on rural and first trimester pregnant women is essential during intervention programs. It is better if researchers explore socio-cultural factors that might inhibit pregnant mothers from receiving the vaccine using a qualitative approach.

## Electronic supplementary material

Below is the link to the electronic supplementary material.

Additional File 1: Questions used to assess knowledge and attitude of pregnant mothers towards COVID-19 vaccine

## Data Availability

The datasets that support the findings of this study are available from the corresponding author upon reasonable request.

## Abbreviations

ANC: Antenatal Care
AOR: Adjusted Odd Ratio
CI: Confidence Interval
COVID-19: Coronavirus Disease 2019
COR: Crude Odd Ratio
DNA: Deoxyribonucleic Acid
RNA: Ribonucleic Acid
WHO: World Health Organization
