# Supplementary material for: Intention to take COVID-19 vaccine and associated factors among pregnant women attending antenatal care at public health facilities in Bahir Dar city, Northwest Ethiopia
Source: BMC Womens Health. 2023 Apr 11;23:175. doi: 10.1186/s12905-023-02331-1 (PMC10088773; doi:10.1186/s12905-023-02331-1)
Supplement: Supplementary file 1 — Additional File 1: Questions used to assess knowledge and attitude of pregnant mothers towards COVID-19 vaccine [file 12905_2023_2331_MOESM1_ESM.docx]

**Questions used to assess knowledge and attitude of pregnant mothers towards COVID-19 vaccine**

|  | **A .Knowledge** | |  |
| --- | --- | --- | --- |
| 101 | Have you heard about COVID-19 vaccine? | 1. 1.Yes 2. 2.No |  |
| 102 | Do you know that COVID19 vaccine is given for pregnant women? | 1. 1.Yes 2. 2.No 3. 3.I don’t know |  |
| 10 | Do you know that the COVID-19 vaccine is available free of cost? | 1. 1.Yes 2. 2.No 3. 3.I don’t know |  |
| 105 | Do you know that the vaccine can decrease the risk of COVID-19 transmission? | 1. 1.Yes 2. 2.No 3. 3.I don’t know |  |
| 105 | Do you know that he vaccine can’t cure already infected people? | 1. 1.Yes 2. 2.No 3. 3.I don’t know |  |
| **B .Attitude** | | | |
| 201 | Do you think that your chance of getting COVID-19 in the next few months is great? | 1. 1.Strong agree 2. 2. Agree 3. 3.Neutral 4. 4.Disagree 5. 5.Strongly Disagree |  |
| 202 | Do you worry about the likelihood of getting COVID 19? | 1. 1.Strong agree   2.Agree   1. 3.Neutral 2. 4.Disagree 3. 5.Strongly Disagree |  |
| 203 | Do you think that getting COVID-19 is currently a possibility for you? | 1. 1.Strong agree 2. 2.Agree 3. 3.Neutral 4. 4.Disagree 5. 5.Strongly Disagree |  |
| 204 | Do you think that Complications from COVID-19 are serious? | 1. 1.Strong agree 2. 2.Agree 3. 3.Neutral 4. 4. Disagree 5. 5.Strongly Disagree |  |
| 205 | Do you think that you will be very sick if you get COVID-19? | 1. 1.Strong agree 2. 2.Agree 3. 3.Neutral 4. 4.Disagree 5. 5.Strongly Disagree |  |
| 206 | Do you afraid of death due to COVID-19? | 1. 1.Strong agree 2. 2.Agree 3. 3.Neutral 4. 4.Disagree 5. 5.Strongly Disagree |  |
| 207 | Do you think that Vaccination is a good idea because you feel less worried about catching COVID-19? | 1. 1.Strong agree 2. 2.Agree 3. 3.Neutral   4. Disagree   1. 5.Strongly Disagree |  |
| 208 | Do you think that Vaccination decreases your chance of getting COVID-19 or its complications? | 1. 1.Strong agree 2. 2.Agree 3. 3.Neutral 4. 4.Disagree 5. 5.Strongly Disagree |  |
| 209 | Do you Worry the possible side-effects of COVID-19 vaccination for your health? | 1. 1.Strong agree 2. 2.Agree   3.Neutral   1. 4.Disagree 2. 5.Strongly Disagree |  |
| 210 | Do you Worry the possible side-effects of COVID-19 vaccination for your fetus? | 1. 1.Strong agree 2. 2.Agree 3. 3.Neutral 4. 4.Disagree 5. 5.Strongly Disagree |  |
| 211 | Do you concern about the efficacy of the COVID-19 vaccination? | 1. 1.Strong agree 2. 2.Agree 3. 3.Neutral 4. 4.Disagree 5. 5.Strongly Disagree |  |
| 212 | Have you concern about the safety of the COVID-19 vaccination? | 1. 1.Strong agree   2. Agree   1. 3.Neutral 2. 4.Disagree 3. 5.Strongly Disagree |  |
| 213 | Do you think that taking COVID19 vaccine interfere with your Religion? | 1. 1.Strong agree 2. 2.Agree 3. 3.Neutral 4. 4.Disagree 5. 5.Strongly Disagree |  |
| 214 | Do you think traditional medicines treat COVID19? | 1. 1.Strong agree 2. 2.Agree 3. 3.Neutral 4. 4.Disagree 5. 5.Strongly Disagree |  |
| 215 | Have you concern of the faulty/fake COVID-19 vaccine? | 1. 1.Strong agree 2. 2.Agree 3. 3.Neutral 4. 4.Disagree 5. 5.Strongly Disagree |  |
| 216 | Do you think that your husband or family support to take COVID19 vaccine? | 1. 1.Strong agree 2. 2.Agree 3. 3.Neutral 4. 4.Disagree 5. 5.Strongly Disagree |  |
| 217 | Will you take the COVID-19 vaccine if you get adequate information about it? | 1. 1.Strong agree   2. Agree   1. 3.Neutral 2. 4.Disagree 3. 5.Strongly Disagree |  |
| 218 | Will you take the COVID-19 vaccine if the vaccine is taken by many in the public? | 1. 1.Strong agree 2. 2.Agree 3. 3.Neutral 4. 4.Disagree 5. 5.Strongly Disagree |  |
